# Physical and Dynamic Oscillatory Shear Properties of Gluten-Free Red Kidney Bean Batter and Cupcakes Affected by Rice Flour Addition

**DOI:** 10.3390/foods9050616

**Published:** 2020-05-11

**Authors:** Pavalee Chompoorat, Napong Kantanet, Zorba J. Hernández Estrada, Patricia Rayas-Duarte

**Affiliations:** 1 Faculty of Engineering and Agro-Industry, Maejo University, Chiang Mai 50290, Thailand; napong250142@gmail.com; 2 Robert M Kerr Food & Agricultural Products Center, Department of Biochemistry and Molecular Biology, Oklahoma State University, Stillwater, OK 74078, USA; zorba.hernandez@itver.edu.mx (Z.J.H.E.); pat.rayas_duarte@okstate.edu (P.R.-D.); 3 Tecnológico Nacional de México/I.T. Veracruz, Calz. Miguel Angel de Quevedo No. 2779 Col. Formando Hogar, Veracruz 91860, Mexico

**Keywords:** dynamic oscillatory shear test, non-isothermal kinetic modeling, gluten-free cupcake, red kidney bean

## Abstract

Red kidney bean (RKB) flour is a nutrient-rich ingredient with potential use in bakery products. The objective of this study was to investigate the viscoelastic properties and key quality parameters of a functional RKB flour in gluten-free cupcakes with different rice flour levels. A 10 g model batter was developed for analyzing the viscoelastic properties of RKB with rice incorporation, in a formula containing oil, liquid eggs, and water. Rice flour was added at five levels 0%, 5%, 10%, 15%, and 25% (*w*/*w*, g rice flour/100 g RKB flour). Rice flour increased RKB batter consistency, solid- and liquid-like viscoelastic behavior and revealed a heterogeneous structure, based on the sweep frequency test. Rice flour at the 25% level increased the shear modulus and activation energy of gelatinization, compared to 0% rice flour addition. Rice flour levels in the RKB batter decreased the inflection gelation temperature from 63 to 56 °C. In addition, the texture of RKB cupcakes with 25% rice flour were 46% softer, compared to the control. The scores from all sensory attributes of cupcakes increased with the addition of rice flour. Rice flour addition improved solid- and liquid-like behavior of the RKB batter and improved the cupcake’s macro-structural characteristics. Overall, 25% rice flour addition performed better than the lower levels. This study confirmed the potential of RKB as a functional ingredient and its improvement in cupcake application with the addition of rice flour.

## 1. Introduction

Red kidney bean (*Phaseolus vulgaris L.*) is a good source of vegetable protein, insoluble and soluble fiber, and is overall a nutrient-rich food ingredient with a great potential for different food applications [1]. Thermal properties, water and oil retention as well as the emulsifying properties of flour of red kidney bean have been reported. Processing including vacuuming and soaking decrease the beany flavor profile of the RKB flour. [2]. However, more studies are needed to fully understand the rheological and sensory properties of the red kidney bean flour and its potential use in bakery products.

Commonly, gluten-free products are low in nutritional value [3,4], with high carbohydrate, low fiber, high glycemic index, as well as saturated and hydrogenated fatty acids [4]. An example of alternative flours and ingredients that increase the protein and bioactive compounds is germinated brown rice flour; a 48 h germination time increases the γ-aminobutyric acid (GABA), polyphenols levels, and antioxidant activity [5]. Unhusked buckwheat flour enhances the fiber content and texture properties of gluten-free bread [6]. However, the use of this ingredient is limited due to its high insoluble fiber from husk, which affects the sensory properties of bread [6]. Rice and tapioca flours are widely used as the main ingredients in gluten-free bakery products due to their neutral color and flavor [7,8]. 

There is an increased interest in using pulses in food ingredients, including FAO declaring 2016 as the International Year of Pulses with the goal of encouraging researchers around the world to investigate more applications of pulses. Pea and chickpea flours have been proposed as alternative flour ingredients in the formulation of gluten-free products [9]. Protein isolates from kidney bean, field pea, and amaranth incorporated in rice-based gluten-free muffins increased the batter viscoelasticity and improved the specific volume and texture [9]. Two types of cowpea protein isolate used in rice flour muffins affected the gluten-free muffin volume differently [10]. The incorporation of the white cowpea protein isolate improved the volume of gluten-free muffin while red cowpea protein isolate decreased the volume [10]. Studies on a mechanistic model to provide a better understanding of baking was addressed by other researchers, including heat and mass transfer during baking and a model for baking of leavened aerated food [11,12,13]. It was proposed that bubble growth during heating showed a lag time before the exponential growth phase, which is related to an expanded inner region of more or less uniform density. The study of rheometric non-isothermal gelatinization kinetics also proposed to model an alteration of gluten-free muffin batter and found that zero-order reaction kinetics can be used to explain batter gelatinization [14]. To the best of our knowledge, the use of the whole red kidney bean flour in gluten-free cupcakes with rice flour has not been investigated. Therefore, the aim of this work was to examine the effect of rice flour on gluten-free RKB batter rheological properties and cupcake quality properties, as well as the interrogation of possible correlations between these properties.

## 2. Materials and Methods

### 2.1. Flour Preparation

Red kidney beans and rice flour were purchased in a local market in Chiang Mai, Thailand. Red kidney beans (RKB) were rinsed, boiled in water for 20 min, strained, and dried for 4 h at 80 °C using a convection oven. These conditions were selected from Chompoorat et al. (2018) [15]. The resulting beans were ground into flour with a hammer mill (W.J. Fit Company, Chicago, IL, USA) to a particle size less than 250 µm (stainless steel sieve # 60, W.S. Tyler Co., Mentor, OH, USA). The RKB flour was stored in polyethylene bags at 4 °C, until needed. RKB flour had 20.5% protein and 14.7% fiber, as previously reported [15]; while, rice flour had 80% carbohydrate, 3.5% protein, and 0.89% fat, according to USDA [16].

### 2.2. Cupcake Preparation

Gluten-free RKB cupcakes were prepared with ingredients purchased from a local market and RKB flour produced in the laboratory. The cupcake formula expressed as a weight percent of RKB flour base (100%) contained granulated white sugar (65%), baking powder (1.9%), baking soda (1.1%), cocoa powder (10%), salt (1%), guar gum (0.5%), vegetable oil (37%), egg (65%), water (150%), and vanilla extract (3%). Commercial rice flour was added at 0%, 5%, 10%, 15%, and 25% based on RKB flour. The control sample contained 100% RKB flour.

The cupcakes were prepared by mixing all dry ingredients (KitchenAid, St. Joseph, MI, USA) for 1 min at the lowest speed (setting# 1), using a paddle attachment. Wet ingredients (vegetable oil, egg, water, and vanilla) were added, mixed for 2 min at low speed (setting #1), followed by 5 min at high speed (setting # 6). Cupcake batter aliquots (40 g) were placed into paper-lined molds and baked in a convection oven at 195 °C for 20 min. The cupcakes were removed from the mold, allowed to cool for 60 min at room temperature, and immediately transferred for analysis of firmness inside a cupcake cardboard box.

### 2.3. Batter Consistency

Batter aliquot (100 g) was used for consistency measurements with a Bostwick consistometer (CSC Scientific Company, Inc., Fairfax, VA, USA), following manufacturer’s directions. The cupcake batter was transferred to the consistometer chamber, the gate was released, and the batter flow rate (cm per min) was recorded. Batter flow rate was inversely proportional to consistency. Measurements were made in three replicates per treatment.

### 2.4. Fundamental Viscoelastic Properties of Batter 

#### 2.4.1. Dynamic Oscillatory Test 

A model batter sample was prepared containing all the ingredients listed in Section 2.2, scaled down to 10 g total. A model batter was freshly prepared for each replicate. The model batter was mixed for 5 min at 100 rpm, using a modified 10-g bowl rotary pin mixer (National Manufacturing, Lincoln, NE, USA). An oscillatory frequency sweep test was based on Hesso et al. (2015) with modifications [17]. An AR-1000N rheometer (TA Instruments, New Castle, DE, USA) was used with a 25-mm plate geometry (cross-hatched to minimize slippage) and a gap of 1 mm at 25 °C. During the test, the plate geometry and batter sample area was covered with a trap to prevent drying or moisture loss. The sample was allowed to recover for 1 min before starting the test. Test conditions included 0.1 to 10 Hz at 0.5% strain, within the linear viscoelastic region. *G′* (storage modulus), *G″* (loss modulus), and tan delta (δ) were determined by this dynamic oscillatory measurement. Analyses were performed in three independent samples.

#### 2.4.2. Temperature-Ramp Test 

A non-isothermal method was applied to study gelation of the batter [18,19,20]. Model batter samples were prepared, as described in Section 2.4.1. Non-isothermal heating of a small amplitude oscillatory shear test (temperature ramp) was measured with an AR-1000N rheometer (TA Instruments, New Castle, DE, USA). A recovering period of 1 min was used before starting the test. Test conditions included a temperature range of 25 to 90 °C, at a heating rate of 5 °C per min, with a constant frequency at 1 Hz and 0.5% strain within the linear viscoelastic region, in which the stress was directly proportional to strain. Analyses were done in three independent samples. Parameters recorded were *G′* (elastic modulus, Pa), the stored energy in each batter sample or recoverable cycle of deformation and *G″* (viscous modulus, Pa), the loss energy in each batter sample or viscous dissipation per cycle of deformation. Complex shear modulus (G*) was calculated (Equation 1, where i represented an imaginary unit) to record the overall resistance to flow and deformation of the samples. G* is a useful property as it is a direct measure of the rigidity of soft solid structure of a material, when exposed to stresses below the yield stress [20,21]. High value of G*, means more solid structure or more resistance to deformation of the samples.

G* =G′ + iG′(1)

The kinetic parameters of the model (Equation (2)) were obtained by numerical methods, according to Alvarez et al. (2017); it was performed with the numerical derivative of the elastic moduli with respect to time. Where *T_0_* temperature (K) at the minimum value of *dG′*/*dt*, a, b, and c are the constants of the model, and c can be used to calculate the *Ea* (kJ.mol^−1^) activation energy (Equation (3)), where *R* is 8.314 J mol^−1^ K^−1^ (the universal gas constant). Activation energy (*Ea*) revealed the energy required for gelatinization [14].

(2) $$\frac{dG^{\prime }}{dt}=a+be^{-c\left(\frac{1}{T}-\frac{1}{T_{0}}\right)}$$

(3) Ea=cR

### 2.5. Firmness of Red-Kidney-Bean–Rice Cupcakes

Cupcake firmness is among the key texture parameters that influence consumer sensory perception and acceptance. After the cupcake samples were cooled to ambient temperature (25 °C), firmness was measured using a TA-XT2 Texture Analyzer (Texture Technologies Corp., Hamilton, MA, USA/Stable Micro Systems, Godalming, Surrey, UK). Analysis was performed following the Approved Method outlined by the American Association of Cereal Chemists (AACC 74-10.02) for bread, using a 25-mm diameter cylindrical probe and 40% compression. Cupcake firmness was performed with 12 independent replicates per sample. 

### 2.6. Sensory Evaluation of Red-Kidney-Bean–Rice Cupcakes

Organoleptic properties of red kidney bean-rice cupcakes were evaluated with a 9-point hedonic scale ranging from ‘dislike extremely’ (1) to ‘like extremely’ (9). Cupcakes were tested after being cooled for 60 min. The samples labeled with random 3-digit codes were scored by twenty untrained panelists, students of the Postharvest Technology at Maejo University, Thailand. The sensory attributes included flavor, taste, texture, and overall quality.

### 2.7. Statistical Analysis

Results are expressed as mean values ± standard deviation of the minimum three independent analyses, unless otherwise described, such as cupcake firmness (*n* = 12) and sensory evaluation (*n* = 20). Mean comparison for significant differences of each treatment was tested by Duncan’s New Multiple Range, using the SAS program (Version 9.1 SAS Institute Inc., Cary, NC, USA). Correlations of the rice flour level effect on batter and cupcake properties were estimated with Principal Component Analysis (PCA) by Canoco for the Windows 5 software (Centre for Biometry, Wageningen, The Netherlands).

## 3. Results and Discussion

### 3.1. Effect of Rice Flour Addition on Red Kidney Bean Batter Consistency

Consistency was estimated with a 100 g-aliquot of full formula batter by measuring the flow rate in a consistometer, as used in the industry for quality control. The test was simple, fast, and provided enough information for consistency standardization. The levels of rice flour significantly decreased the flow rate (increased consistency) of gluten-free RKB cupcake batter, starting at 10% rice flour addition (Table 1). Rice flour addition of 10%, 15%, and 25% significantly decreased the flow rate of the gluten-free cupcake batter by 4%, 12%, and 20%, respectively, compared to the control sample. Data fitted a second-degree polynomial (y = −0.0022x^2^−0.0659x + 14.163, *R*^2^ = 0.955) reflecting negative slopes starting at 10% rice level. Increased consistency of the batter matrix could be explained in part by the increase in air entrainment forming air bubbles, which increase the elastic character of the batter [22]. This might have been favored by rice flour properties and specifically of rice starch. We speculated that the continuous viscous phase of batter containing rice flour has more organized hydrogen bonds with water molecules, resulting in increased consistency, as observed by a decreased flow rate. A study of rice flour and starch showed that water-binding capacity of rice flour was higher than rice starch, which were 128% and 100%, respectively [23]. The value of rice was within the range of red kidney bean flour, with 125% water absorption [24]. Starch damage in the rice flour might also have contributed to an increased water-holding capacity [25,26]. The results agreed with reports of a flow rate study of rice flour and wheat flour, which proposed that a decrease in flow rate in batter was due in part to the rate of flour water absorption [27,28]. Previous studies have showed that amylose content is negatively correlated with water absorption, swelling power, and damaged starch [29,30,31]. Rice flour has amylose content approximately 15% lower than red kidney bean flour [32,33]. Thus, this low amylose content of rice flour could impact the consistency of batter.

### 3.2. Effect of Rice Flour Addition on Red Kidney Bean Batter Viscoelastic Properties

#### 3.2.1. Frequency Sweep Test of Batter

**Figure 1:**
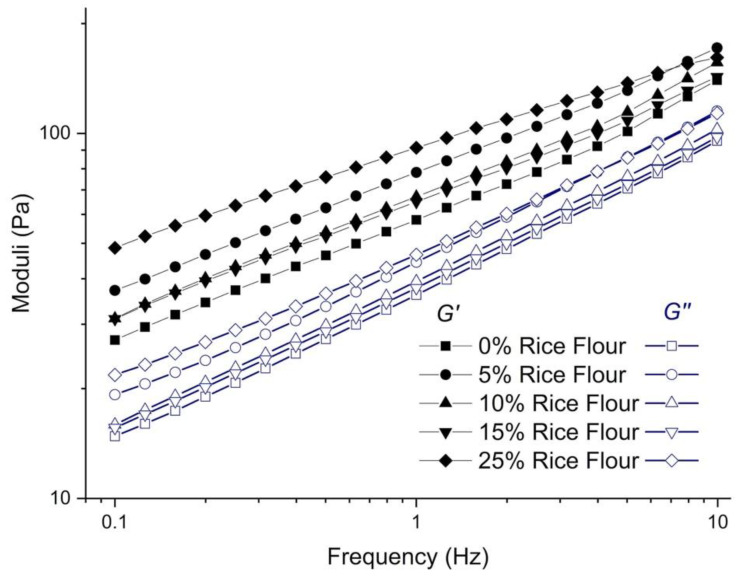
Moduli (*G′* and *G″*) as a function of frequency of red kidney bean gluten-free batter with different rice flour levels.

*G′* (storage modulus) and *G″* (loss modulus) increased as the levels of rice flour increased in the frequency range of 0.1 to 10 Hz (Figure 1). Elastic behavior dominated in all gluten-free RKB cupcake batter samples (*G′* > *G″*). Cake batter is considered a wet foam with contributions to its bulk rheology from the continuous viscous phase and the discontinuous bubble phase (elastic effect) introduced during mixing and the production of gas from leavening agents [22]. At 1 Hz and 0.5% strain, tan δ of the batter with 25% rice addition decreased by 18%, compared to the control (data not shown), suggesting that the solid character (stiffness) of the batter increased by 18%. From Figure 1, a 2.3 fold increase in the solid-like behavior (*G′*) of the batter (22.4 Pa for control up to 50.7 Pa for 25% rice flour) was higher than the 1.3 fold increase of the liquid-like behavior (*G″*) (17.6 Pa for control and up to 22.1 Pa for 25% rice flour) (Figure 1). Adequate viscosity is a factor contributing to the containment of the bubbles. Structural visualization studies are needed to identify the distribution of the starch (specifically the separation of small and large starch granules), proteins, and fat, as well as the size of the bubbles in the batter. Hesso et al. (2015) reported that air incorporation increased with an increase in batter viscosity [17]. It is also postulated that the rice starch might align in the interphase of oil in the batter [23]. The results are in overall support of the increase in consistency of the full formula batter described in Section 3.1, as it decreased the flow rate after rice addition. In agreement to this study, a report of rice-based gluten-free batter for muffins with different levels of cowpea protein isolates found that the elastic behavior increased more than the viscous behavior [25]. In our study, enhanced viscoelasticity of the RKB gluten-free batter with increased levels of rice flour suggested the formation of composite matrix structures with enhanced water-binding capacity, as mentioned earlier (Section 3.1). We speculated that a decrease in free water availability for other ingredients, mainly protein and fiber that form more disorganized structures, would result in a more organized matrix. The levels of rice flour in the RKB batter system improved the gel rigidity and the network of RKB batter [7]. In addition, the results showed a solid-like behavior (*G′*) for the batter; when the rice flour content varied from 5% to 15%, the rice flour showed a similar behavior. 

**Figure 2:**
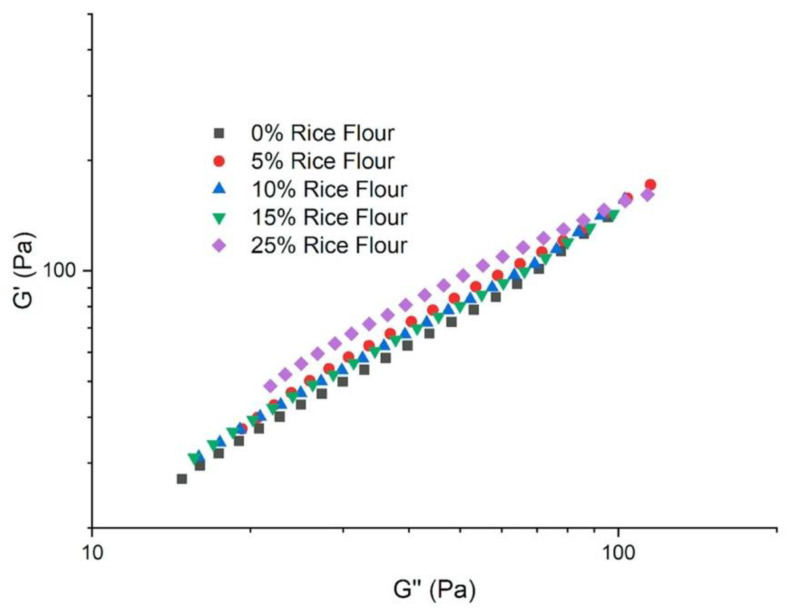
Log–log plot of G′ as a function of G″ of red kidney bean gluten-free batter with different rice flour levels.

Figure 2 depicted the log–log plots of *G′* and *G″* that were used to evaluate if the elastic and storage moduli followed a power law relationship in the RKB batter samples, with different rice flour levels. If the response was linear, then the relationship followed a power law, suggesting that a constant increase of *G′* led to a constant increase in *G″*. The same plot, known as the Han plot, was used to determine the compatibility of polymer blends [34,35]. Overall, the separation of the curve containing 25% rice flour from the control RKB flour was observed as a shift to higher elasticity (*G’*), compared to the control (*p* = 0.05) (Figure 2). The tracings of 0% to 15% rice converged, suggesting that they are compatible. At the terminal area, all the curves merge (0% to 25% rice flour), suggesting that at a higher frequency, the lines would have similar correlation. [35]. The 25% rice flour curve separates by shifting to a rise in elasticity compared from the other rice levels, suggesting the formation of more heterogeneous structures compared to the other samples, through the polymers present in the flours [36,37]. The increased values for *G′* with *G′* in 25% rice flour compared to other levels might be a contribution of the multiple structures, where less protein and fiber from the RKB flour and overall more starch from the rice flour formed a new continuous network with a higher organization (lower enthalpy), as compared to the control RKB flour and other rice levels. Our previous study of RKB batter with different heat-moisture treatments showed that the high temperature altered the viscoelastic properties of the RKB batter; however, their structures were homogenous [15]. Thus, Figure 2 confirmed the heterogeneous nature of the RKB batter containing rice flour. 

#### 3.2.2. Temperature Ramp Test of Batter

**Figure 3:**
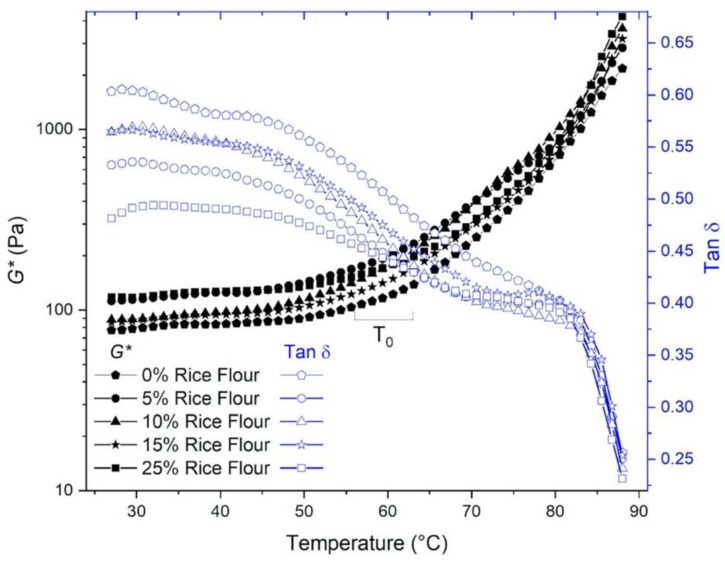
Complex modulus G* (left axis) and tan (δ) (right axis) as a function of the temperature of the red kidney bean gluten-free batter with different rice flour levels. Filled symbols, complex modulus G*; and open symbols tan δ.

The viscoelastic properties of the RKB batter with and without rice during heating from 25 °C to 90 °C with constant values of frequency and strain indicated that the complex shear modulus (G*) increased as the tan δ decreased (Figure 3). The almost linear response of the complex shear modulus changed to an upward curve after the gelation onset, indicating an increase in gel rigidity during heating, which reached up to a 94% increase (at 90 °C) with 25% rice flour, compared to the starting temperature of 25 °C. RKB batter with different rice flour levels showed overall similar rising gel rigidity (G*) trends after 50 °C (Figure 3) suggesting similar viscoelastic patterns, during gelatinization. 

The tracing curves of tan δ showed two (rice flour additions) and three (RKB flour) clear inflection points, suggesting different changes of structures (phase transitions) as a function of increasing temperature (Figure 3). Rice flour at 25% had a significantly (*p* = 0.05) lower tan δ at the initial stages of heating before the gelation onset, compared to the rest of the samples, suggesting a higher solid-like character (*G′*) in this sample. That higher solid-like behavior was conserved up to around 50 °C, where the decrease of tan δ accelerated, suggesting a rapid increase in solid-like behavior. This was attributed to the starch and protein hydration, resulting in gel rigidity onset. Tan δ curves crossed-over the G* curves at around 60 °C (rice flour additions) and 65 °C (RKB flour control), suggesting more rapid changes occurring in the structures. Most likely these changes were related to the denaturation of proteins and the rapid starch granule swelling at those temperatures [29,30]. The predominant formation of hydrogen bonds with water and additional hydrophobic interactions from protein aggregation and starch swelling account for the increase G* and decrease tan δ (Figure 3), in the continuous phase of the batter. 

Activation energy (*E_a_*) was used as an indication of the energy used for the critical gel rigidity during gelatinization, while the inflection temperature (*T_0_*) was used for the point at which the batter viscoelasticity increased at the initial stage of gel point. *T_0_* is normally associated with batter density and level of gelatinization [14]. Initiation of gelatinization in all RKB batter samples was in the range of 56 to 63 °C (Table 1). These observations agreed with literature reports where onset temperature variation during starch gelatinization from four kidney bean cultivars was in the range of 61.4–66.9 °C, while the onset temperature for rice starch gelatinization was 61 °C, measured by differential scanning calorimetry (DSC) [38,39]. Reported gelatinization range (70 to 85 °C) for basmati rice starch measured by an increase in *G′* was higher than the values of our study [39]. Increase in rice flour content decreased the RKB batter inflection gelation temperature (*T_0_*) from 63 to 56 °C, with no significant (*p* = 0.05) change in the activation energy (*E_a_*). Significant difference (*p* = 0.05) in activation energy was observed only between the control RKB flour and 25% rice addition, where a 44% increase was needed to reach the critical gel rigidity (Table 1). Activation energy is related to phase transitions during gelatinization of starch, glass transition of starch granules in the amorphous region, and melting transition in the crystalline region [39]. Thus, the addition of rice flour induced gelation at lower temperatures and the process required a higher activation energy, only when rice flour was added at 25% (*p* < 0.05). This could be partially explained by the smaller starch granule size of rice flour (3–8 µm), compared to the kidney bean starch (16–60 µm), increase in overall starch content in the system, and RKB flour protein reduction [32,33]. Literature reports for rice and kidney bean flour gelatinization enthalpy, individually, are in a similar range 7.5–15.6 J/g for rice flour and 10.8–15.4 J/g for kidney bean flour, respectively [40,41]. Activation energy (*Ea*) from this non-isothermal heating of small amplitude oscillatory shear test could be useful to indicate the changes during gelatinization.

### 3.3. Effect of Rice Flour Addition on Red Kidney Bean Cupcake Firmness

Cupcake texture estimated by firmness is one of the most important parameters for consumer acceptance. As the level of rice flour increased, the RKB cupcakes firmness decreased, reaching a 45.5% reduction with 25% rice flour, compared to the control (Table 1). These observations agreed with a reduced (50% lower) firmness of a rice-based, gluten-free muffin, compared to muffins containing white cowpea protein isolate at 12% level [10]. A decrease in cupcake firmness might be attributed to the factors discussed earlier including the smaller starch granule size of rice, compared to RKB and different molecular organization of rice, lowering the overall protein content in cupcakes, combined with a redistribution of water availability in the gluten-free cupcake. Water absorption and protein content were 2.1 and 2.8 times higher in RKB flour, compared to rice flour (2.6 and 1.23 g/g and 20.5% and 7.4%, for RKB and rice flour, respectively) [15,40]. The incorporation of rice flour changed the structural order of the batter by diluting the proteins and lowering the firmness of gluten-free RKB rice cupcake. Amylose content in rice (average 25%) is lower than kidney bean flour (34% to 41%) and would result in less retrogradation after baking [33,40]. Thus, diluting the amylose content of kidney bean flour by rice flour addition reduced gluten-free cupcake hardness. Previous studies showed that an increase in batter viscosity allowed gas entrapped within the batter structure. This resulted in higher cake volume, crumb with increased airy structure, thinner walls, and thus lower firmness [42,43]. 

### 3.4. Effect of Rice Flour Addition on Red Kidney Bean Cupcake Sensory Properties

Rice flour impacted the organoleptic properties of red kidney bean cupcake, with higher sensory scores observed with the samples containing rice flour. The red-kidney-bean–rice cupcakes were scored from ‘like slightly’ to ‘like moderately’ (Table 2). There was no change in the sensory scores at the 5% rice flour level. Scores for flavor and taste attributes of the red-kidney-bean–rice cupcakes were higher when rice flour was added at 10%. All attributes had a highest score with 25% rice flour. It was interesting to note that the sensory panelists did not observe changes in texture until 25% rice flour level, when they scored the cupcakes higher, while the overall quality score was improved at 15% rice flour. The nutritional value of the RKB cupcakes of 100 g serving size included 404 Kcal, 18 g total fat, of which 4 g were saturated fat, 104 mg cholesterol, 187 mg sodium, 48 g total carbohydrates, 9 g dietary fiber, 27 g sugar, and 14 g of protein. No significant differences in nutritional value were observed with addition of rice flour at up to 25%.

### 3.5. Variation of Red Kidney Bean Batter and Cupcake Properties with Rice Flour Addition

**Figure 4:**
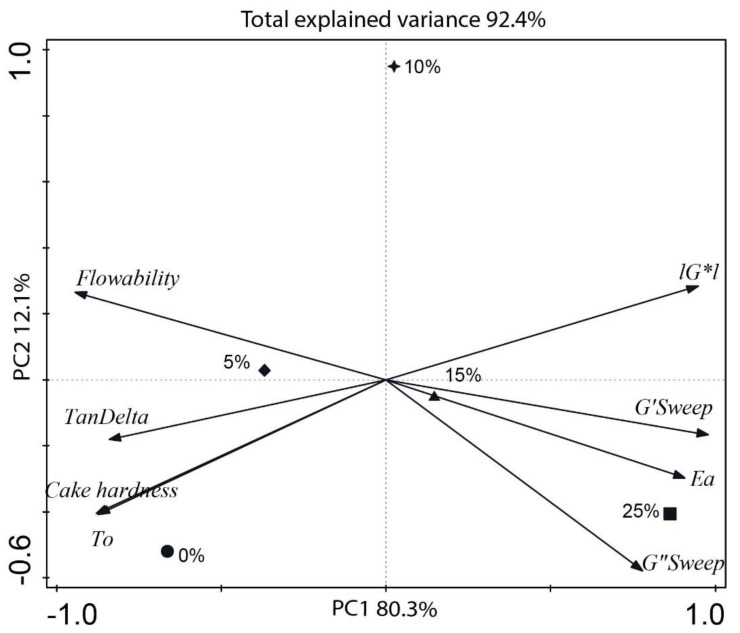
Redundancy analysis of red kidney bean gluten-free batter with and without rice flour with 8 indicators of the viscoelastic properties of gluten (definitions in Table 1), showing the effect of five rice flour levels (0%, 5%, 10%, 15%, and 25%). Symbols: 0%—circle, 5%—diamond, 10%—star, 15%—up triangle, and 25%—square.

Total explained variance from all variables was 92.4% (Figure 4). Principal component 1 (PC1) had the majority of explained variance (80.3%), while the contribution of principal component 2 (PC2) was only 12.1% (Figure 4). The main contributors of PC1 (first coordinate axis) were batter complex shear modulus, activation energy of gelatinization, and tan δ. The main contributor to PC2 was the sample with 10% rice addition—situated almost at the top, at 90 degrees, at the center of the graph. However, only one parameter (*G’’* viscous behavior) moved slightly to the horizontal PC2, meaning it had some variations explained by it. The fact that 10% rice addition was almost in a direction opposite to the viscous behavior also suggest that it was inversely correlated to 10% rice addition. However, as the contribution to the variance was low—only 12.1%—compared to 80.3% contribution to the variance of PC1, it was better to highlight the contributions of PC1. Notice how all parameters were along PC1. The vectors aligned in opposite direction revealed negative correlation, while the vectors in the same direction were positively correlated. The results from the loading plot (Figure 4) revealed that cupcake firmness was positively correlated with batter consistency, tan δ (TanDelta in the graph) and inflection temperature (*T_0_*) of gelation, and the treatments associated with those parameters were 0% and 5% rice levels. Complex modulus G*, solid (*G′*) and viscous (*G″*) moduli were positively correlated with the activation energy (*Ea*) of gelatinization and the highest rice flour level (25%), which was the farthest away from the center of the graph. Rice flour addition to RKB flour was negatively correlated to cupcake firmness. These results confirmed that the most variation was observed at the highest rice level (25%). This study also revealed that the gelation of the batter and its consistency were good indicators of quality control for gluten-free RKB–rice flour cupcakes. 

## 4. Conclusions

The level of rice flour addition improved the viscoelastic properties of gluten-free RKB batter and cupcake softness. With 25% of rice flour addition in gluten-free RKB flour, the system increased the batter’s solid-like and viscous behavior, batter consistency, inflection of gelatinization and temperature, and produced a softer cupcake texture. The RKB batter with rice followed a power law and had a heterogeneous structure based on the log–log plot of *G′* vs *G″*. In addition, the activation energy of gelatinization increased with 25% rice incorporation. This study described an improvement of the RKB batter and cupcake macro-structural characteristics with addition of rice. 

## Figures and Tables

**Table 1:** Batter and cupcake characteristics of gluten-free red kidney bean flour with rice flour addition ^a,b^.

Rice Flour Level ^c^ (%)	Batter Characteristics			Cupcake Characteristic
	Consistency	*T_0_*	*Ea*	Firmness
	(cm/min)	(°C)	(kJ mol^−1^)	(g)
**0**	14.3 ± 0.3 ^a^	63.0 ± 2.5 ^a^	165 ± 3 ^b^	2970 ± 5 ^a^
**5**	14.0 ± 0.1 ^a^	60.2 ± 2.3 ^b^	191 ± 2 ^a,b^	2767 ± 4 ^a^
**10**	13.2 ± 0.3 ^b^	56.8 ± 2.5 ^c^	177 ± 2 ^b^	1709 ± 3 ^b^
**15**	12.3 ± 0.3 ^c^	56.7 ± 2.3 ^c^	206 ± 4 ^a,b^	1871 ± 3 ^b^
**25**	11.2 ± 0.3 ^d^	56.0 ± 2.5 ^c^	237 ± 2 ^a^	1619 ± 3 ^b^

^a^ Consistency (flow rate), Temperature ramp test: *T_0_*, inflection temperature of gelation and *Ea*, activation energy of the batter measured as flow rate. *Firmness*, cake texture. ^b^ Mean values (*n* = 3 ± SD) followed by a different letter within a column are significantly different, Duncan test (*p* < 0.05). ^c^ Rice flour addition to red kidney bean flour.

**Table 2:** Sensory properties for red-kidney-bean–rice cupcakes ^a,b^.

Rice Flour Level (%) ^b^	Flavor	Taste	Texture	Overall Quality
0	6.1 ± 0.1 ^b^	6.2 ± 0.2 ^b^	6.2 ± 0.5 ^b^	6.1 ± 0.1 ^b^
5	6.2 ± 0.2 ^b^	6.3 ± 0.1 ^b^	6.1 ± 0.2 ^b^	6.0 ± 0.2 ^b^
10	7.1 ± 0.3 ^a^	7.2 ± 0.2 ^a^	6.2 ± 0.1 ^b^	6.1 ± 0.1 ^b^
15	7.3 ± 0.4 ^a^	7.1 ± 0.0 ^a^	6.1 ± 0.2 ^b^	7.1 ± 0.1 ^a^
25	7.1 ± 0.3 ^a^	7.1 ± 0.2 ^a^	7.1 ± 0.1 ^a^	7.2 ± 0.2 ^a^

^a^ Mean values (*n* = 20 ± SD) followed by a different letter within a column are significantly different, Duncan test (*p* < 0.05). Evaluated by a 9-point hedonic scale ranging from ‘dislike extremely’ (1) to ‘like extremely’ (9). ^b^ Rice flour level addition to red kidney bean flour.
